# Correction: Topological phase transition in monolayer 1T′-MoS_2_

**DOI:** 10.1038/s41598-025-09567-x

**Published:** 2025-07-01

**Authors:** Mohammad Mortezaei Nobahari, Mahmood rezaei Roknabadi

**Affiliations:** Department of Physics, Ferdowsi University of Mashhad, Mashhad, Iran

Correction to: *Scientific Reports* 10.1038/s41598-025-01593-z, published online 10 May 2025

The original version of this Article contained an error in the spelling of the author Mohammad Mortezaei Nobahari which was incorrectly given as Mohammad Mortezaie Nobahari.

The original Article has been corrected.

